# Machine learning-based early diagnosis of autism according to eye movements of real and artificial faces scanning

**DOI:** 10.3389/fnins.2023.1170951

**Published:** 2023-09-15

**Authors:** Fanchao Meng, Fenghua Li, Shuxian Wu, Tingyu Yang, Zhou Xiao, Yujian Zhang, Zhengkui Liu, Jianping Lu, Xuerong Luo

**Affiliations:** ^1^The National Clinical Research Center for Mental Disorder & Beijing Key Laboratory of Mental Disorders, Beijing Anding Hospital, Capital Medical University, Beijing, China; ^2^Department of Psychiatry, and National Clinical Research Center for Mental Disorders, The Second Xiangya Hospital of Central South University, Changsha, Hunan, China; ^3^Key Lab of Mental Health, Institute of Psychology, Chinese Academy of Sciences, Beijing, China; ^4^Department of Child Psychiatry, Kangning Hospital of Shenzhen, Shenzhen Mental Health Center, Shenzhen, Guangdong, China; ^5^Sichuan Cancer Hospital & Institute, Sichuan Cancer Center, Chengdu, Sichuan, China

**Keywords:** autism spectrum disorder, eye-tracking, cartoon character, machine learning, random forest

## Abstract

**Background:**

Studies on eye movements found that children with autism spectrum disorder (ASD) had abnormal gaze behavior to social stimuli. The current study aimed to investigate whether their eye movement patterns in relation to cartoon characters or real people could be useful in identifying ASD children.

**Methods:**

Eye-tracking tests based on videos of cartoon characters and real people were performed for ASD and typically developing (TD) children aged between 12 and 60 months. A three-level hierarchical structure including participants, events, and areas of interest was used to arrange the data obtained from eye-tracking tests. Random forest was adopted as the feature selection tool and classifier, and the flattened vectors and diagnostic information were used as features and labels. A logistic regression was used to evaluate the impact of the most important features.

**Results:**

A total of 161 children (117 ASD and 44 TD) with a mean age of 39.70 ± 12.27 months were recruited. The overall accuracy, precision, and recall of the model were 0.73, 0.73, and 0.75, respectively. Attention to human-related elements was positively related to the diagnosis of ASD, while fixation time for cartoons was negatively related to the diagnosis.

**Conclusion:**

Using eye-tracking techniques with machine learning algorithms might be promising for identifying ASD. The value of artificial faces, such as cartoon characters, in the field of ASD diagnosis and intervention is worth further exploring.

## Introduction

Social interaction impairment is the most common clinical manifestation of autism spectrum disorder (ASD), which is characterized by verbal and nonverbal communication difficulties as well as stereotyped obsessive behaviors (Volkmar et al., 2004). Abnormal eye contact during social situations is among the most noticeable manifestations of social interaction difficulties for those with ASD (Leekam et al., 1998; Spezio et al., 2007). Early screening remains one of the major challenges in ASD research. According to a recent meta-analysis involving 30 studies with over 60,000 ASD participants from 35 countries, the age for diagnosis occurred at approximately 60 months of age, which was late for early intervention to be initiated (Van’t Hof et al., 2021). Delayed diagnosis and intervention will have a negative impact on children’s prognoses and may lead to lifelong unpleasant outcomes, posing a significant burden on families and society.

Tremendous efforts have been made by clinical workers to create techniques for the early screening of ASD, and there are numerous tools available (Sappok et al., 2015). However, due to the popularity of existing instruments and incorrect operating methods, some missed diagnoses may occur in places with exceptionally large populations and few or no community health workers (James et al., 2014). The results have aroused the attention of professionals involved in the early detection of ASD.

Eye-tracking technology has become an increasingly important tool in the early screening and diagnosis of ASD in recent years. In contrast to electroencephalography (EEG) and magnetic resonance imaging (MRI), which are time-consuming and difficult to perform, eye-tracking is regarded as a very child-friendly tool that enables a variety of original designs for the investigation of visual exploration patterns and their underlying mechanisms. Evidence proved that eye-tracking techniques combined with machine learning algorithms might be promising in the early and objective diagnosis of ASD (Kollias et al., 2021). Because of ASD children’s difficulties in social interaction, the complexity of social interaction is lacking, and eye-tracking technology can capture the distinctions between high and low social significance stimulation in ASD children via the stimulation paradigm. Studies investigating the factors influencing social attention in ASD found a decrease in gaze to stimuli with high social significance and an increase in gaze to stimuli with low social significance (Chita-Tegmark, 2016a; Frazier et al., 2017). For example, there was decreased attention to the entire face and upper face regions, increased attention to body regions and other unimportant or extraneous aspects of stimuli, and decreased attention to the lower face (mouth) (Chita-Tegmark, 2016b; Frazier et al., 2017).

The total time of gaze fixation with low social significance (such as geometric figures) has been successfully applied as a criterion to distinguish ASD (Shi et al., 2015; Pierce et al., 2016; Moore et al., 2018). Multiple studies for ASD identification using machine learning with eye-tracking data exhibited accuracies of 67–98% in non-toddler groups (Kollias et al., 2021). To the best of our knowledge, there were no studies combining eye tracking using cartoons as stimuli with machine learning algorithms. Using cartoons as stimuli has several advantages. First, it might better capture the attention of toddlers who can be easily influenced by the outside environment, especially when they are not interested in the proposed stimuli (Masedu et al., 2022). Second, a recent study found that ASD children had lower levels of social orientation (SO) than TD children in the realistic task but comparable levels in the cartoon task. Nonetheless, their findings indicated that the cartoon task effectively captured developmental and adaptive delay by demonstrating numerous correlations with visual exploration parameters such as social prioritization, fixation duration, and percentage of SO (Robain et al., 2022). In addition, studies investigating factors that influence social attention in ASD found that ASD children seem to process cartoon faces in a similar manner that typical development (TD) children do; they tend to look more at cartoon characters than at other objects in cartoon situations (Van der Geest et al., 2002). The differences between these groups make it simpler for us to capture the complexities of ASD social interaction and then infer the difference between ASD and TD children in social interaction, which becomes a diagnostic signal. Therefore, given that developmental delay and abnormal gaze are early markers of ASD, we hypothesized that the cartoon task, as well as other minimally social stimuli, could be a useful tool for early screening.

## Methods

### Participants

Participants were recruited between 2019 and 2021 in Changsha and Shenzhen, China. The inclusion criteria for children with ASD were as follows: (1) those aged 12–60 months; (2) those who met the diagnosis of ASD according to the Diagnostic and Statistical Manual of Mental Disorders, fifth edition (DSM-V) (American Psychological Association, 2009) and the Autism Diagnostic Observation Schedule (ADOS) confirmed the diagnosis (Lord et al., 1999); and (3) children with normal vision and hearing who can complete the eye movement tests. Those with other major mental disorders or serious physical health problems were excluded. Toddlers who participated when they were younger than 24 months old were classified as global developmental failures based on their performance on the Chinese version of the Gesell development scale (GDS) (Yang, 2016). They were followed and diagnosed every 3–6 months until they were 2 years old. TD children aged 12–60 months were recruited without gender restrictions. According to their parents/caregivers, they had no evidence of developmental disabilities or neuropsychiatric conditions.

The research was carried out in accordance with the Declaration of Helsinki’s ethical principles. The experimental procedures had been explained to all participants’ parents or caregivers, and written informed consent was obtained from all of them. The ethics committee of the Second Xiangya Hospital, Central South University, reviewed and approved the study (No. 2017YFC1309904).

### Clinical assessments

#### Autism diagnostic observation schedule

ADOS is a semi-structured, standardized observational tool that is frequently used as a diagnostic indicator for ASD. It can accurately assess and diagnose ASD using a variety of play-based activities that focus on communication, social engagement, play, and innovative use of materials, as well as restricted and repetitive behaviors (González et al., 2019).

#### Chinese version of GDS

GDS was used to assess the development of children. It is composed of five domains: adaptability, gross motor, fine motor, language, and social–emotional responses (Yang, 2016). Participants’ development quotient (DQ) in each domain was calculated. Using the full-scale DQ, the development was classified as normal (DQ ≥ 85), deficient (DQ ≤ 75), or borderline (75 ~ 85). In this field, DQ in any single domain less than 75 was considered deficient.

### Eye-tracking acquisition and processing

The eye-tracking tests were carried out in a quiet environment. A SensoMotoric Instruments Red500 remote eye tracker (Teltow, Germany) was attached to the frame of a 1,680 × 1,050 22-inch LCD stimulus presentation monitor. The highest spatial resolution and gaze position accuracy were 0.1 and 0.4, respectively. The capture range for eye movement was 40° horizontally (±20°) and 60° vertically (±40°). The tracking range of the head motion is 40 × 20 cm when the man–machine distance is 70 cm. Throughout the experiment, two 5-point calibrations were obtained at fixed times.

**Figure 1:**
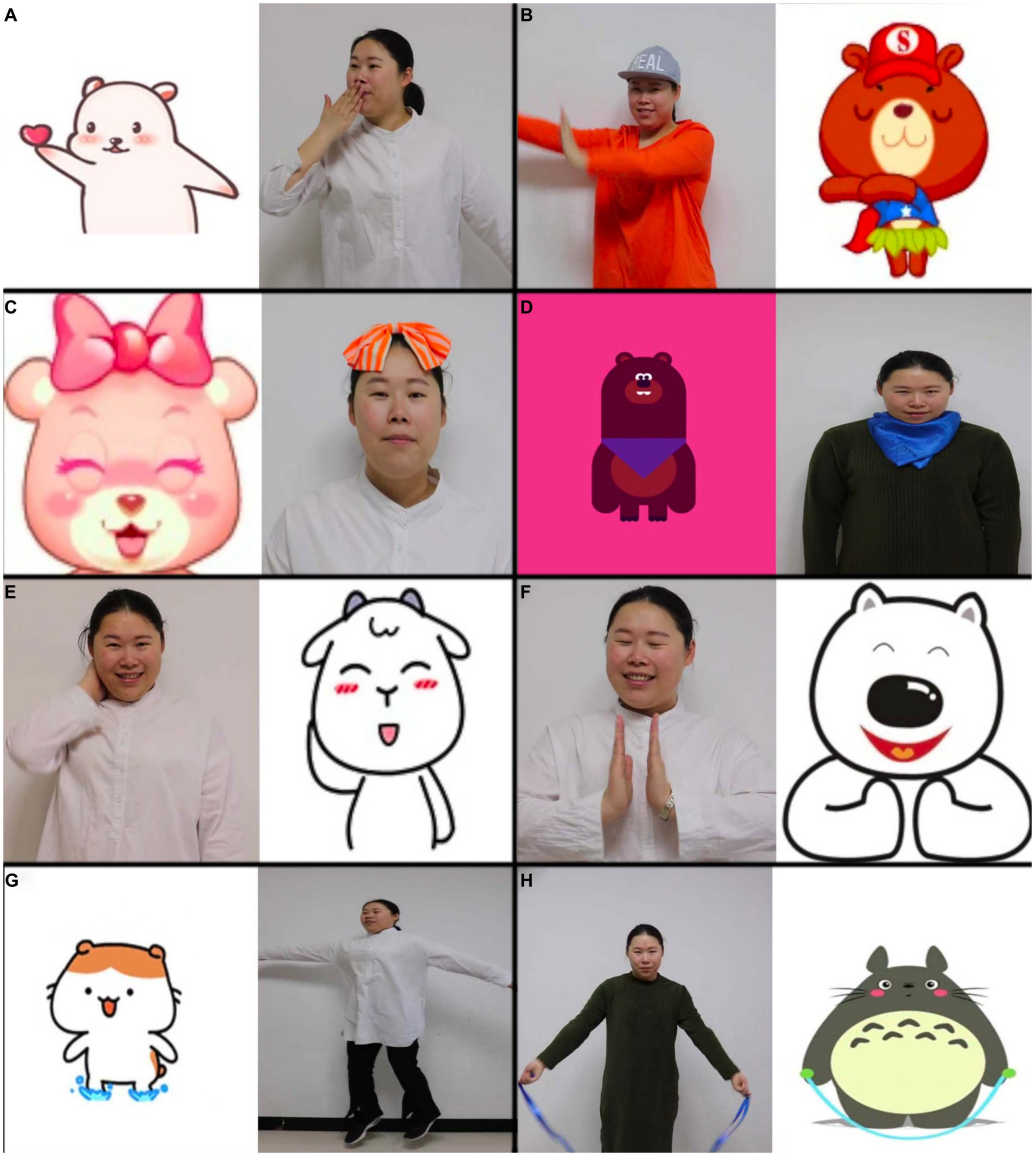
Pictures of eight videos used for eye-tracking tests, **(A-H)** represented different scenarios.

Eight videos were played in the SMI Experiment Center. Each video has two large rectangular areas side by side, and the screen is also divided into the left and right sides. There was a cartoon character on the left (or right) side playing actions such as dancing, nodding, blowing a kiss, scratching the neck, clapping hands, bouncing, skipping rope, and nodding while stretching thumbs. The opposite side presented a real person. This person imitates the cartoon character. During the imitation process, the person tried to match the expression (smiling or no obvious expression), movement, clothing (clothing color and accessories), character size, and appearance (half body or whole body) with the cartoon pattern (Figures 1A–H). All of the videos were soundless, and the SMI Experiment Center software was created using a random playback option so that each child would see the eight videos in a different order.

### Data organization

**Figure 2:**
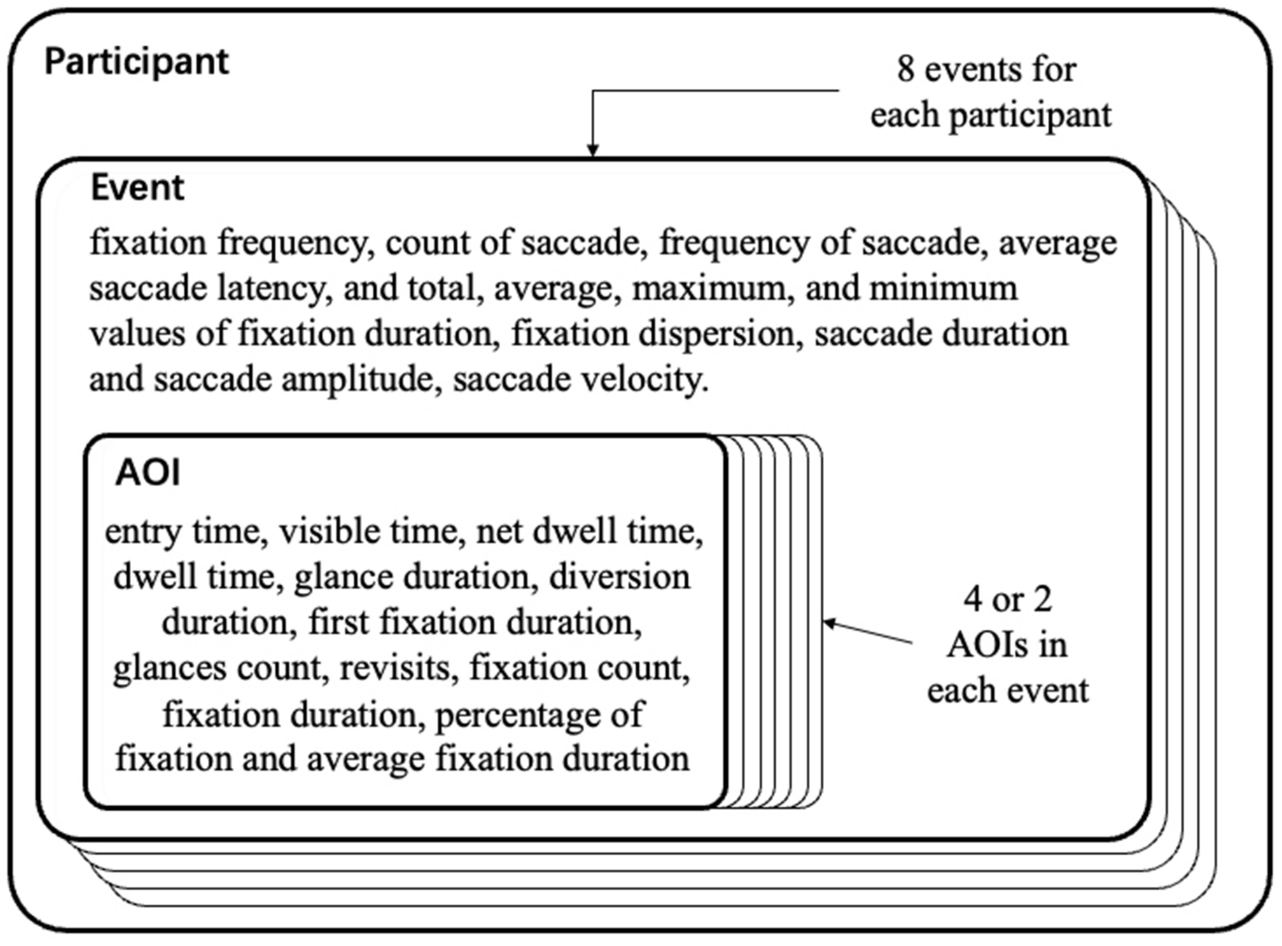
Three-level hierarchical structure and data item of each level.

**Figure 3:**
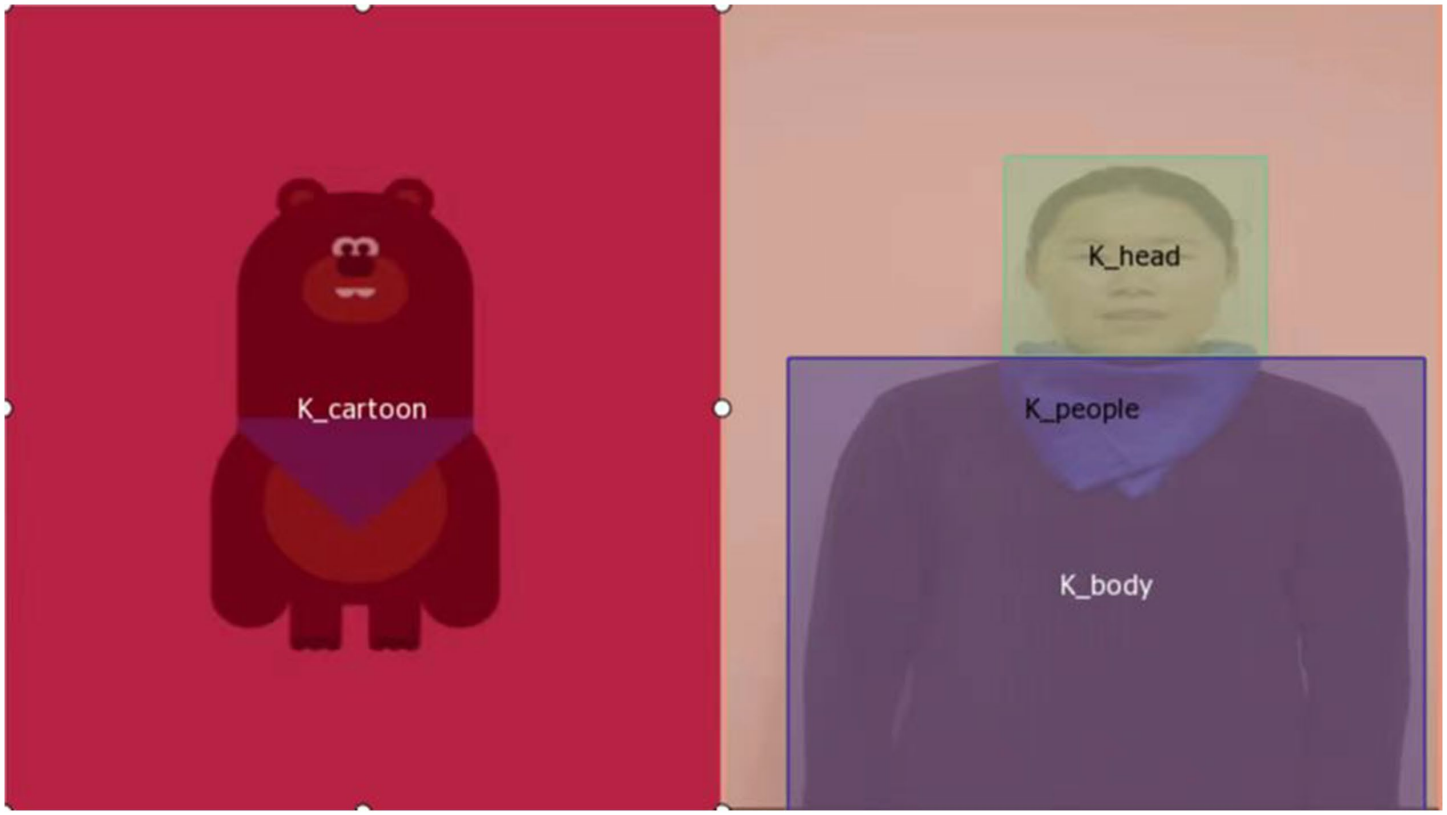
The four area of interests in the three events.

**Figure 4:**
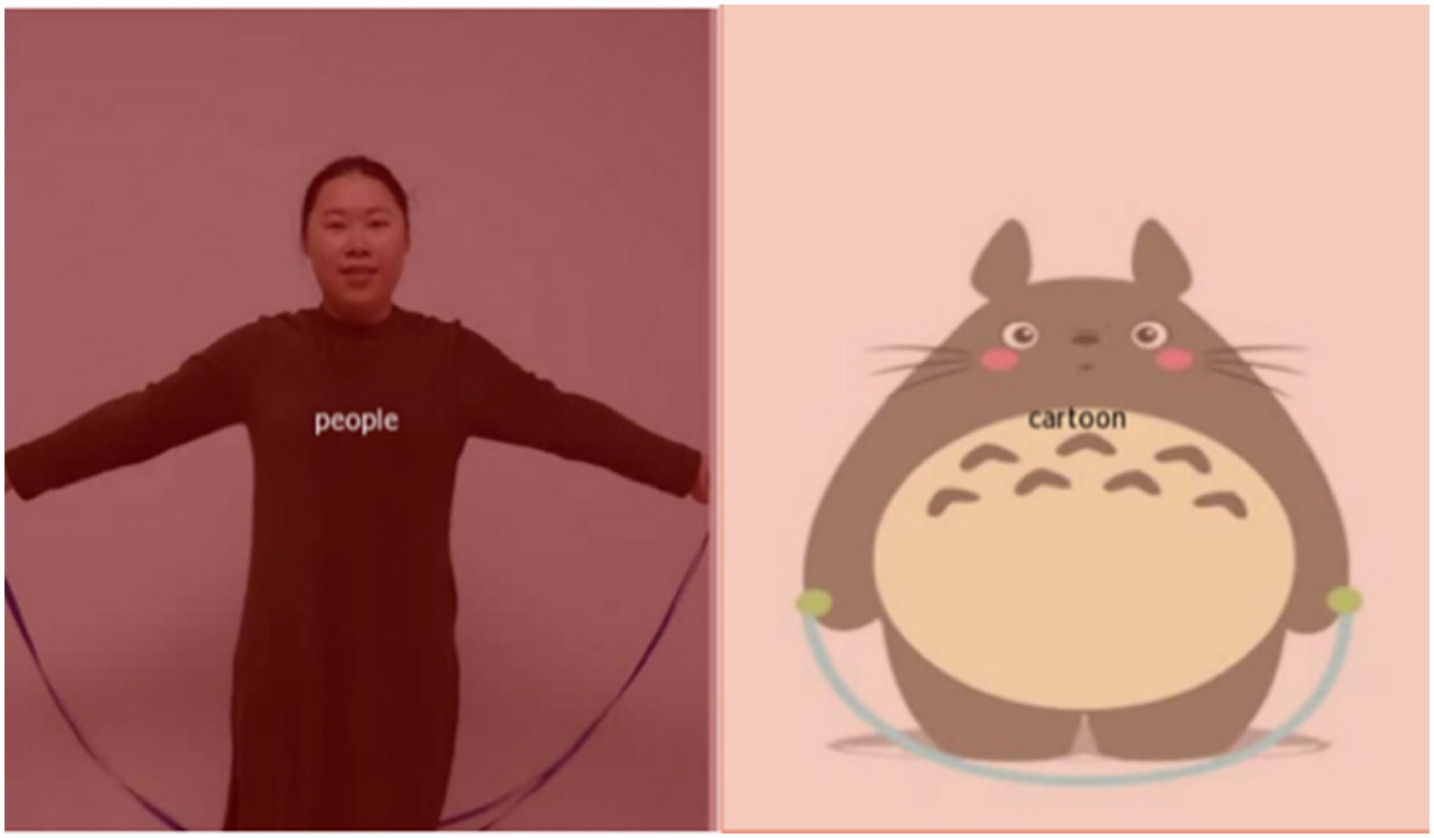
The two area of interests in the five events.

A three-level hierarchical structure [participants, events, and area of interest (AOI)] was used to arrange the data obtained by the SMI BeGaze program (Figure 2). Each participant had eight event data entities, matching one of the eight videos used in the trials. A total of 24 data items, including fixation frequency, saccade amplitude, count, frequency, average latency, fixation dispersion, saccade length, saccade velocity, and total, average, maximum, and minimum values of fixation time, were recorded for each event data entity. Three events (nodding, clapping hands, and nodding while stretching thumbs) had four AOIs (cartoon, people, people’s heads, and people’s bodies) (Figure 3). Five events (dancing, blowing a kiss, scratching the neck, bouncing, and skipping rope) had two AOIs (cartoon and people) (Figure 4). Each AOI contained a set of 14 data items. The 14 data items within each AOI data entity were as follows: entry time, visible time (equivalent to the duration of the event in this study), net dwell time (time of all gazes that hit the AOI), dwell time (sum of net dwell time and time of saccades that hit the AOI), glance duration (sum of dwell time and duration of saccade entering the AOI), and diversion duration (sum of glance duration and duration of saccade leaving the AOI). In this study, gaze refers to the non-saccade movement status, and fixation refers to a cluster of gaze points that are close in space and time (60 ms).

### Data analysis

**Figure 5:**
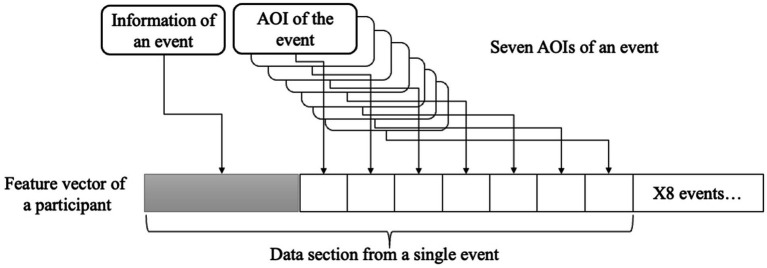
A feature vector consisted of flattened data structure of a participant.

We flattened each participant’s hierarchical data structure into a single vector with a length of 688 elements in order to thoroughly investigate the data items collected from the participants. Therefore, an array of event items and AOI entities from a single participant were arranged consecutively (Figure 5). Flatten vectors and diagnostic information were used as features and labels, and random forest (RF) was used as the feature selection method and classifier.

RF is a common ensemble classifier and feature-selection technique (Menze et al., 2009; Boulesteix et al., 2012; Biau and Scornet, 2016). Multiple independent decision trees make up an RF. Each decision tree uses a random subset of the input features from a random subset of the training examples to fit itself during the training phase. The predictions of all the fitted trees were averaged to determine the final classification of an RF. In addition to the final choice, an RF also produces a significant value for each feature (entropy decrease, Gini impurity, etc.).

In our research, we created equal-sized autistic and healthy groups using the undersampling technique and constructed RF models. Gini impurity was utilized as the split criterion, and there were 800 decision trees. The ratio of training to validation cases was 7:3. The average accuracy, precision, and recall were obtained after 500 iterations of the fitting process (Powers, 2020). Additionally, we plotted the receiver operating characteristic curve (ROC) using all the prediction findings and determined the area under the curve (AUC). The undersampling and training-validation splits were randomized before each of the 500 fitting processes.

A logistic regression was applied to examine if the features were related to the diagnoses. Forward stepwise factor selection was used to build the logistic regression model. Features in the RF models were used as independent variables in the regression model. Features were added to the model one at a time. A feature was chosen if statistical significance could be found in the feature itself or if the statistical significance of previously added features was unaffected by the new feature. Python 3.8.10, sci-kit learn 1.1.1, and the R language 4.1.1 were used for all data organization and analysis.

## Results

The characteristics of the participants are presented in Table 1. A total of 161 children with a mean age of 39.70 ± 12.27 months were recruited, and 47 of them were girls. Among them, 117 were diagnosed with ASD and 44 with TD. There were 91 boys and 26 girls in the ASD group and 23 boys and 21 girls in the TD group. The children in the ASD group were significantly younger than those in the TD group. Children in the ASD group had significantly lower scores in the GDDS compared to their healthy controls.

**Table 1:** Clinical characteristics of participants.

Characteristics	ASD (*N* = 117)	TD (*N* = 44)	Value of *p*
Female, *n* (%)	26 (22.2)	21 (47.7)	<0.001
Age, mean (SD), months	38.7 (12.8)	46.0 (11.9)	0.002
Adaptive behavior, mean (SD)	65.7 (16.8)	94.0 (9.5)	<0.001
Gross motor, mean (SD)	73.4 (15.2)	89.4 (14.6)	<0.001
Fine motor, mean (SD)	71.23 (19.5)	92.0 (8.9)	<0.001
Language, mean (SD)	46.9 (19.4)	98.7 (15.7)	<0.001
Personal–social behavior, mean (SD)	59.1 (18.1)	101.6 (10.1)	<0.001

ASD, autism spectrum disorder; SD, standard deviation; TD, typically developing.

**Figure 6:**
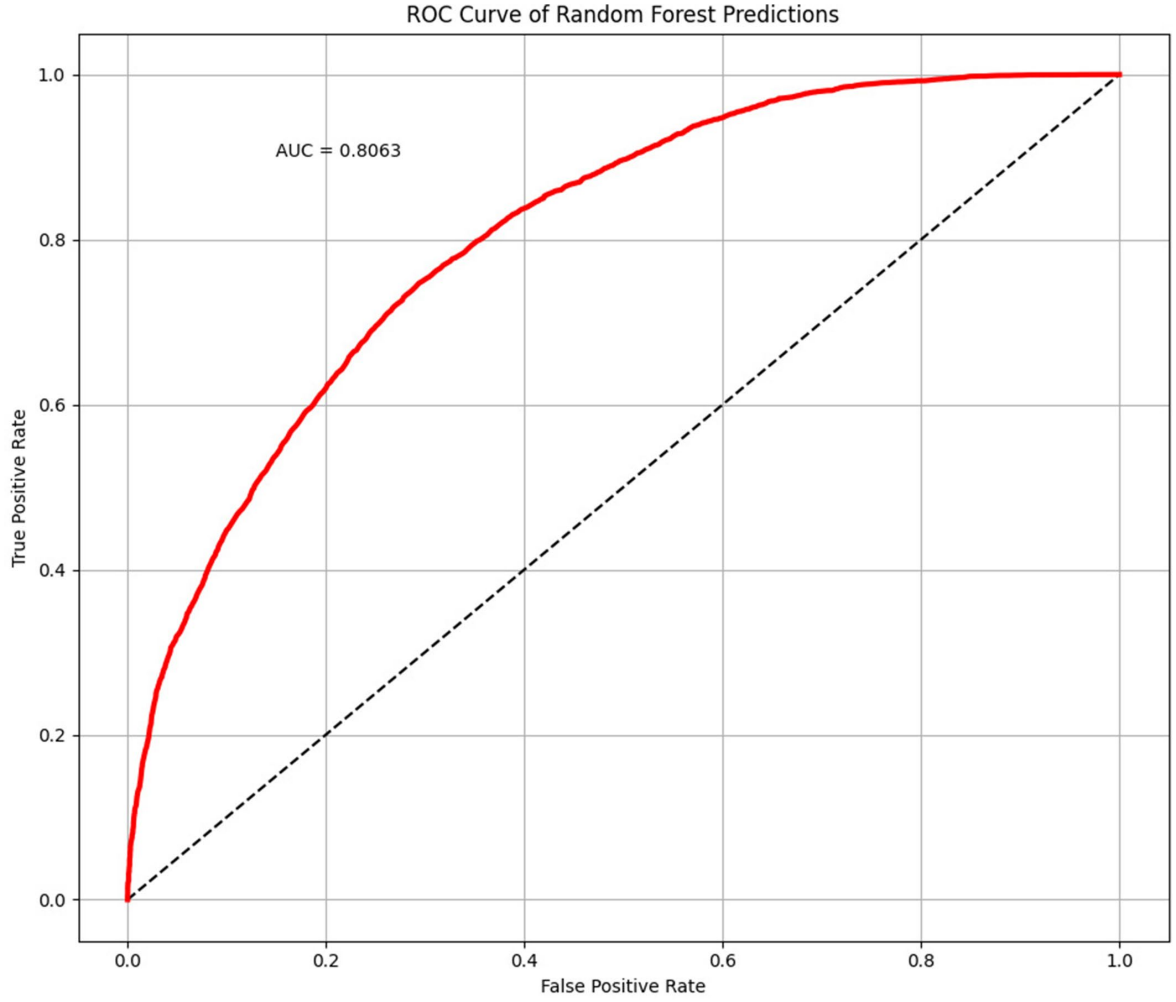
Receiver operating characteristic (ROC) curve and area under curve (AUC) plotted with 500 times randomized validation results.

The average accuracy, precision, and recall of the 500-time fitting validation were 0.73, 0.73, and 0.75, respectively, and the AUC was 0.81 (Figure 6). Randomized undersampling and the train-test split were performed before every fitting. The sizes of the ASD and TD groups were both 44 after undersampling. The size ratio of the training and validation sets was 7:3.

In the logistic regression analysis, we first included age as the independent variable before the stepwise factor selection as children in the ASD and TD groups had significantly different ages. In addition to age, the best-fit logistic model found that nine features were significantly associated with an ASD diagnosis (Table 2). The features that were negatively associated with ASD were body-revisits and head-glance-duration when the cartoon character makes the gesture of clapping hands, cartoon-sequence when scratching the neck, people-glances-count when nodding while stretching thumbs, and people-net-dwell-time when dancing. The features that were positively associated with ASD were people-first-fixation-duration when the people are dancing, cartoon-sequence when the character is nodding, and cartoon-fixation-time when blowing a kiss. In addition, the saccade velocity maximum has a positive correlation with ASD diagnoses (Table 2).

**Table 2:** The best-fit logistic regression model.

Features	Estimate	Std. error	*z* value	Value of *p*
(Intercept)	1.22	0.22	5.55	<0.001
Clapping hands: body-revisits	−0.41	0.20	−2.07	0.038
Clapping hands: head-glance-duration	−0.59	0.23	−2.52	0.012
Dancing: people-first-fixation-duration	1.04	0.27	3.80	0.000
Scratching the neck: cartoon-sequence	−0.62	0.21	−2.95	0.003
Nodding while stretching thumbs: people-glances-count	−0.95	0.24	−3.98	<0.001
Nodding: cartoon-sequence	0.54	0.21	2.58	0.010
Saccade velocity maximum	0.52	0.21	2.42	0.015
Blowing a kiss: cartoon-fixation-time	0.63	0.22	2.82	0.005
Dancing: people-net-dwell-time	−0.63	0.25	−2.54	0.011

## Discussion

In this study, we developed a novel ASD identification framework for children using a specific cartoon paradigm, together with eye movement data for cartoon preferences and machine learning. We gave children a task based on Pierce et al. (2011), in which videotaped moving/dancing kids were pitted against geometric patterns moving repetitively. The cartoon and real human figures were used at the same time in the stimulus design to make sure the clothing and actions in the cartoon were comparable to those in the real figures. ASD and TD children had different visual preferences for cartoon and real human faces, and ASD children preferred cartoon faces much more than TD children (Van der Geest et al., 2002; Rosset et al., 2008). According to the results, our stimulus paradigm had satisfactory efficacy in distinguishing ASD from TD children. The ML model has an average accuracy, precision, and recall ASD of 0.73, 0.73, and 0.75, respectively. As a classifier to differentiate between ASD and TD children, it has an AUC of 0.81.

Similar efforts have been made by some other studies combining eye-tracking technology with ML algorithms for the objective diagnosis of ASD. For example, Liu et al. (2016) used a data-driven approach to extract features from face scanning data, and a support vector machine (SVM) was applied in the data analysis. While this study showed a maximum classification accuracy of 88.5%, it had a relatively small sample size with 29 ASD children included. Kang et al. (2020) recruited 77 low-functioning autistic children and 80 TD children to watch a random sequence of face photos. With SVM, they found a maximum classification accuracy of 72.5% (AUC = 0.77). However, all of their participants were aged between 3 and 6 years, and no younger children were included. Consistent with previous findings (Tao and Shyu, 2019; Tsuchiya et al., 2021), our study has the advantage of including the largest sample size of younger children under the age of three. Other researchers promote the research ideology that early diagnosis and special education can be accomplished through the use of computer-aided methods based on EEG signals and/or imaging; however, the results in different studies are not quite the same. Wee et al. (2014) reported the greatest accuracy of 96% using SVM as a classifier in a study utilizing sMRI (structural magnetic resonance image). Haar et al. (2016) conducted a large-scale investigation with 245 ASD and 245 control subjects, using cortical surface area as a feature and linear discriminant analysis as a classifier, and reported a low accuracy of 60%. Rane et al. (2017) conducted the largest fMRI study, with over a thousand participants. A low accuracy rate of 60.56% was obtained. Wang et al. (2019) had over a thousand participants as well and reported a higher accuracy of 93.59%. Although the research findings are highly exciting, we prefer to use eye movement in clinical promotion because the clinical operability of MRI is significantly more challenging.

In the current study, we found several glancing behaviors that related to real humans or human parts, such as body-revisits, head-glance-duration, people-glance-count, and people-net-dwell-time, were all negatively associated with ASD. In contrast, glancing behaviors that related to cartoon characters such as cartoon-sequence and cartoon-fixation-time were positively associated with ASD. These behaviors had social significance. Children were more likely to have ASD when they were less interested in real humans or human parts and were more interested in cartoon characters. These results were similar to previous findings that adults with ASD were slow to respond to social stimuli, especially when there were non-social stimuli competing with social stimuli that were related to the narrow interests of ASD (Sasson and Touchstone, 2014; Wang et al., 2014). People with a higher degree of autistic features showed a greater interest in non-human social beings such as animals, robots, or cartoons (Atherton and Cross, 2018). One possible explanation for the cartoon preference of ASD children is that cartoons do not require social interaction. That is to say, the typicality of ASD in cartoon processing may be due to the damage to their social communication skills (Rosset et al., 2008).

Notwithstanding, we found that people-first-fixation-duration when dancing was positively associated with an ASD diagnosis. The reason under this might be that real people had a greater range of motion and children were more attracted to this motion. Another unexpected result was that when watching the video of scratching the neck, ASD children showed quick attention to the cartoon area, while when watching the video of nodding, they showed slow attention to the cartoon area. We compared these two videos and found that in the video of nodding, the face size of the cartoon character was significantly larger than that of a real human. This result reminds us that although cartoon characters have lower social intensity than real people, ASD children also showed similar face avoidance when the face size was relatively large (Falck-Ytter and von Hofsten, 2011). This study utilizes non-linear machine learning models to select a series of indicators, which have been automatically summarized through multiple iterations of machine learning. These indicators possess strong data-driven characteristics and are not heavily reliant on the specific features of the selection method itself. According to our statistics, the predictive capabilities of each indicator decrease with the order of the indicator list. However, it is essential to note that this research utilizes a non-linear machine learning method (RF) for selection. While the authors attempted to provide explanations for the rationality of the selected indicators, their individual use or linear combination to construct predictive models may not necessarily achieve the same effect as when combined in the RF. This is determined by the working mechanism of the RF’s decision trees, where the same indicator may be used multiple times based on different premises at different decision nodes.

There are several limitations to this study. First, the artificial undersampling may lead to an increase in false-positive judgments in an ecological setting, especially when the prevalence is extremely low. Nonetheless, the samples collected in this study differ significantly from the real-world prevalence of ASD (117 positive cases and 44 negative cases). Therefore, without the use of undersampling to balance the samples, it would not be possible to create a model that better adapts to large-scale screening. Moreover, an abundance of positive samples might mislead the model, which is another reason why we ultimately decided to use balanced samples. In future research, these extracted indicators should be applied to fit a screening model more suitable for ecological settings in larger-scale samples (such as screening studies at the provincial level). Second, the age and development level among ASD and TD children were different, and these differences might affect the face scanning patterns (Yi et al., 2014). We did not consider age in the prediction model, and we just focused on whether there was a discrepancy in task performance. With a balanced age distribution and cognitive levels, incorporating age range as a factor in the model would further improve its performance. However, due to the limitations of sample size and an imbalanced age distribution, this study has not been able to achieve this step. Third, though we found a correlation between eye movement features and ASD diagnosis, considering the complex nonlinear classification characteristics of RF, the actual eye movement patterns of ASD children were still not fully clear to us, especially when facing cartoon characters. Fourth, even though our study included the largest sample size of children under the age of three, a larger sample size is still needed in future studies. In particular, those who are at high risk of ASD and younger than 2 years old are needed to validate our results and model.

Despite these limitations, the current study demonstrated that using eye-tracking techniques with ML algorithms might be promising for identifying ASD. The value of artificial faces, such as cartoon characters in the field of ASD diagnosis and intervention is worth further exploring.

## Data availability statement

The raw data supporting the conclusions of this article will be made available by the authors, without undue reservation.

## Ethics statement

The studies involving humans were approved by the ethics committee of the Second Xiangya Hospital, Central South University, reviewed and approved the study (No. 2017YFC1309904). The studies were conducted in accordance with the local legislation and institutional requirements. Written informed consent for participation in this study was provided by the participants’ legal guardians/next of kin.

## Author contributions

FM, SW, TY, ZX, and YZ performed the experiments. FL and ZL performed the statistical analysis. FM revised the manuscript. JL drafted the manuscript. XL designed the study. All authors have given final approval for the version to be published.

## Funding

This work was supported by Youth Talent Training “Green Seedling Program of Beijing Hospital Management Center” (No. QML20231906 to FM), Shenzhen Fund for Guangdong Provincial High-level Clinical Key Specialties (No. SZGSP013 to JL), National Key R&D Program of China (No. 2017YFC1309900 to XL), and Key Research and Development Program of Hunan Province (No. 2019SK2081 to XL).

## Conflict of interest

The authors declare that the research was conducted in the absence of any commercial or financial relationships that could be construed as a potential conflict of interest.

## Publisher’s note

All claims expressed in this article are solely those of the authors and do not necessarily represent those of their affiliated organizations, or those of the publisher, the editors and the reviewers. Any product that may be evaluated in this article, or claim that may be made by its manufacturer, is not guaranteed or endorsed by the publisher.
